# These Shoes Are Made for Walking: Sensitivity Performance Evaluation of Commercial Activity Monitors under the Expected Conditions and Circumstances Required to Achieve the International Daily Step Goal of 10,000 Steps

**DOI:** 10.1371/journal.pone.0154956

**Published:** 2016-05-11

**Authors:** Sandra O’Connell, Gearóid ÓLaighin, Lisa Kelly, Elaine Murphy, Sorcha Beirne, Niall Burke, Orlaith Kilgannon, Leo R. Quinlan

**Affiliations:** 1 Discipline of Physiology, School of Medicine, NUI Galway, Galway, Ireland; 2 Electrical & Electronic Engineering, School of Engineering & Informatics, NUI Galway, Galway, Ireland; 3 National Centre for Biomedical Engineering Science, NUI Galway, Galway, Ireland; 4 CÚRAM, Center for Medical Device Research, NUI Galway, Galway, Ireland; IRCCS E. Medea, ITALY

## Abstract

**Introduction:**

Physical activity is a vitally important part of a healthy lifestyle, and is of major benefit to both physical and mental health. A daily step count of 10,000 steps is recommended globally to achieve an appropriate level of physical activity. Accurate quantification of physical activity during conditions reflecting those needed to achieve the recommended daily step count of 10,000 steps is essential. As such, we aimed to assess four commercial activity monitors for their sensitivity/accuracy in a prescribed walking route that reflects a range of surfaces that would typically be used to achieve the recommended daily step count, in two types of footwear expected to be used throughout the day when aiming to achieve the recommended daily step count, and in a timeframe required to do so.

**Methods:**

Four commercial activity monitors were worn simultaneously by participants (n = 15) during a prescribed walking route reflective of surfaces typically encountered while achieving the daily recommended 10,000 steps. Activity monitors tested were the Garmin Vivofit ^™^, New Lifestyles’ NL-2000 ^™^ pedometer, Withings Smart Activity Monitor Tracker (Pulse O_2_) ^™^, and Fitbit One ^™^.

**Results:**

All activity monitors tested were accurate in their step detection over the variety of different surfaces tested (natural lawn grass, gravel, ceramic tile, tarmacadam/asphalt, linoleum), when wearing both running shoes and hard-soled dress shoes.

**Conclusion:**

All activity monitors tested were accurate in their step detection sensitivity and are valid monitors for physical activity quantification over the variety of different surfaces tested, when wearing both running shoes and hard-soled dress shoes, and over a timeframe necessary for accumulating the recommended daily step count of 10,000 steps. However, it is important to consider the accuracy of activity monitors, particularly when physical activity in the form of stepping activities is prescribed as an intervention in the treatment or prevention of a disease state.

## Introduction

Physical activity is an essential part of a healthy lifestyle, playing an important role in improving and maintaining both physical and mental health [1–4]. Indeed, lack of physical activity has been recognized as the fourth leading risk factor for global mortality, associated with 6% of deaths worldwide [5]. Since the benefits of physical activity have been recognized globally, governments internationally have highlighted to their citizens the need to be physically active on a regular basis. In 1996, the US Surgeon General recommended that as part of a healthy lifestyle, people of all ages should partake in at least 30 minutes of moderate-intensity physical activity, such as brisk walking, on a daily basis [6]. The 2008 ‘Physical Activity Guidelines for Americans’ from the Centers for Disease Control (CDC) recommended that adults need 2 hours and 30 minutes of moderate intensity aerobic physical activity, for example brisk walking, per week [7]. These two recommendations correspond closely with the 2010 WHO recommendation that adults should do at least 150 minutes of moderate intensity aerobic physical activity throughout the week [5].

An important consideration in assisting persons to change their physical activity behavior and to adhere to these public health recommendations is providing them with the ability to easily quantify the extent of physical activity completed in a given day. The development of commercial pedometers in the late 1960s provided a convenient, low cost method of quantifying one form of physical activity, namely walking. These devices provided straightforward feedback to the user of the quantity of physical activity completed, specifically their step count. A series of pedometer-based studies, from 2004 to 2011, evaluated the relationship between step count and adherence to physical activity guidelines and reported that less than 7,500 steps per day represented sedentary or “low-active” behavior [8, 9] and taking 10,000 steps per day was consistent with a physical activity level associated with person who is “active” [8, 10, 11]. Furthermore, a study carried out in an overweight population from the Lower Mississippi Delta of the U.S.A. reported a step count of 9,154 steps per day to equate to 30 minutes of moderate-to-vigorous physical activity. The authors concluded that, in this population, a step count of 8,300 to 9,100 steps a day should be accumulated in order to achieve recommended physical activity guidelines [12].

As part of their physical activity guidelines, the WHO states that physical activity in adults includes recreational or leisure-time physical activity, occupational (i.e. work), household chores, transportation (e.g. walking or cycling), play, games, planned exercise or sports in the context of daily, family, and community activities [5]. Thus the recommended “quantity” of physical activity of 10,000 steps being proposed internationally would only typically be achieved through a combination of these activities and under the varied circumstances associated with these activities. In other words, the recommended step count of 10,000 steps would typically be achieved, not only through a specific programme of exercise, but also through a combination of activities in the home, in the work place, and while getting to and from work. These activities would typically involve the person walking on a wide variety of surfaces from footpaths, indoor floors with varied surface types, and outdoor natural walking surfaces such as grass pathways. Indeed, one group have examined the effect of walking surface on step count sensitivity/accuracy of a pedometer and found surface type significantly affected step count [13]. Additionally, a number of physical activity device manufacturer websites state that surface type can affect step count sensitivity/accuracy of their devices [14, 15].

Furthermore, the range of activities conducted in achieving the recommended step count of 10,000 steps would also typically involve the person changing their footwear from more formal “dress” shoes in the workplace to more comfortable runner-type shoes in the home and while exercising. Therefore, a physical activity monitor being used to quantify adherence to the recommended daily step count must be able to deal with: (i) a variety of walking surfaces, (ii) different footwear, and (iii) extended periods of walking, where the accumulated number of steps is in the order of 10,000 steps.

These three variables have the potential to affect step count detection accuracy. Thus, it is the view of the authors that activity monitor testing must involve: (i) walking on different surfaces that reflect the range of surfaces that will typically be encountered to achieve the daily step count goal, (ii) walking with different footwear that reflects the different types of footwear expected to be used throughout the day, and (iii) walking for periods of time that properly reflect the time required to achieve the recommended daily step count. In fact, a review of the literature highlights that these considerations have not been widely adopted in a wide range of studies where the sensitivity of activity monitors has been assessed [16–21]. Often, the types of surfaces and types of footwear tested are either not reported or not taken into consideration during testing. Furthermore, the timeframe of testing is often far less than would be required to achieve the recommended daily step count of 10,000 steps. For example, timeframes of between 11 and 25 minutes have previously been utilized and would be completely insufficient for accumulating 10,000 steps [16–20].

In this paper we aimed to assess four commercial activity monitors: the Withings Smart Activity Monitor Tracker (Pulse O_2_) ^™^, the NL-2000 pedometer ^™^, the Garmin Vivofit ^™^, and the Fitbit One ^™^. We aimed to assess the step detection sensitivity of each activity monitor, i.e. the ability of the activity monitors to detect and count an actual step as a step, over a prescribed walking route that reflects a range of surfaces that would typically be used to achieve the recommended daily step count and in a timeframe required to do so. In addition, we also aimed to investigate two different types of footwear typically used throughout the day when aiming to achieve the recommended daily step count of 10,000 steps.

## Methods

### Participants

Fifteen healthy participants (8 female, 7 male) took part in this study, with a mean age of 21.1 ± 1.1 years. Males had a mean BMI of 23.60 ± 2.70kg/m^2^ while females had a mean BMI of 21.88 ± 1.81kg/m^2^. None of the participants had any history of cardiovascular disease or neurological disorders. Ethics committee approval was obtained from the Galway University Hospitals Research Ethics Committee, and all participants provided written, informed consent.

### Study Protocol

**Fig 1:**
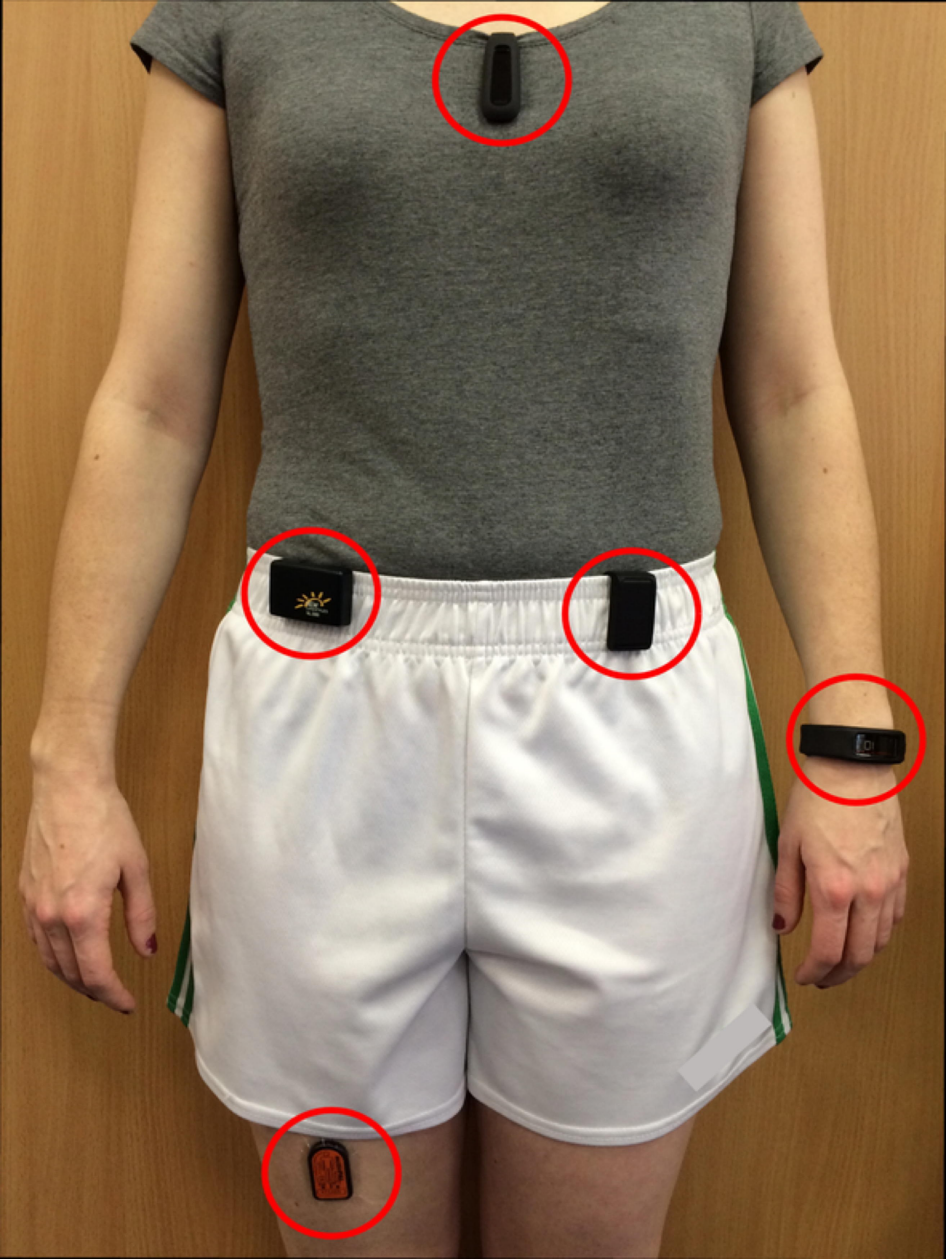
Position of the activity monitors. The ActivPAL micro ^™^ activity monitor on the thigh (right), the NL-2000 ^™^ pedometer on the waist (right), the Garmin Vivofit ^™^ on the wrist (left), the Withings Smart Activity Monitor Tracker (Pulse O_2_) ^™^ on the waist (left), the Fitbit One ^™^ activity monitor on the chest.

All 15 participants completed a prescribed walking route. The activity monitors tested in this study were the Withings Smart Activity Monitor Tracker (Pulse O_2_) ^™^ (Withings, Issy-les-Moulineaux, France), NL-2000 pedometer ^™^ (New Lifestyles, Missouri, USA), Garmin Vivofit ^™^ (Garmin, Kansas, USA), and Fitbit One ^™^ (Fitbit, San Francisco, USA). (See Table 1). All activity monitors were put in place at the manufacturer’s recommended body location (Fig 1) by the investigators as per the manufacturers’ instructions. All four activity monitors were worn simultaneously on each participant for the duration of testing. The Garmin Vivofit ^™^ was worn on the non-dominant wrist, with the NL-2000 ^™^ and Withings Smart Activity Monitor Tracker (Pulse O_2_) ^™^ worn on opposite sides of the waist. The Fitbit ^™^ activity monitor was clipped onto clothing at the level of the chest. Participants were video recorded throughout the study with a hand-held camcorder. The true step count was extracted manually from the recorded video in real time and was then compared to the step count registered by each activity monitor. Additionally, the ActivPAL micro ^™^ (PAL Technologies Ltd., Glasgow, UK) was worn by each participant as a reference device for measuring the overall total step count for the prescribed walking route. The ActivPAL ^™^ was attached to the thigh using Tegaderm transparent dressing (3M Health Care, Minnesota, USA).

**Table 1:** Details of the different activity monitors tested.

Activity Monitor	Specifications	Outputs	Interface	Location of attachment	Validation Studies
ActivPAL micro ^™^	*Sensor*: 3-axis accelerometer. *Recording/ battery life*: 10+ days	Step count; time sitting/lying, standing, stepping; number of transitions from standing to sitting; energy expenditure	Need to connect to PC to download and view data	Anterior aspect of the thigh	Ryan et al., 2006 [22]; Godfrey et al., 2007 [23]; Maddocks et al., 2008 [24]; Grant et al., 2008 [25]; Busse et al., 2009 [26]; Harrington et al., 2011 [27]; Storm et al., 2015 [19]
Withings Smart Activity Monitor Tracker (Pulse O_2_) ^™^	*Sensor*: 3-axis accelerometer optoelectronics sensor. *Recording/ battery life*: 2 weeks	Step count; distance; elevation; calories; sleep cycle; heart rate & blood O_2_	Screen and smart phone application	On the wrist (detachable wristband); clipped on at the hip (detachable clip); in a pocket	Ferguson et al., 2015 [28]; Storm et al., 2015 [19]
Fitbit One ^™^	*Sensor*: 3-axis accelerometer; altimeter. *Recording/ battery life*: 10–14 days	Step count; distance; elevation; calories; sleep cycle	Screen and smart phone application	Clipped on at hip or chest level; in a pocket; in a wristband (at night)	Ferguson et al., 2015 [28]; Storm et al., 2015 [19]; Klassen et al., 2015 [29]; Simpson et al., 2015 [30]; Diaz et al., 2015 [31]; Lee et al., 2014 [32]; Tackas et al., 2014 [20]
Garmin Vivofit ^™^	*Sensor*: 3-axis accelerometer. *Recording/ battery life*: 24/7 recording; more than one year battery life	Step count; distance; calories; sleep cycle; heart rate; sitting, standing	Screen and smart phone application	-Wristband	
NL-2000 pedometer ^™^	*Sensor*: piezoelectric strain gauge. *Recording/ battery life*: 7 day automatic memory; removable 3V lithium battery	Step count; elevation; calories	Screen	-Clipped on at the hip level	Crouter et al., 2003 [33]; Schneider et al. 2003 [34]; Schneider et al., 2004 [35]; Grant et al., 2008 [25]

**Fig 2:**
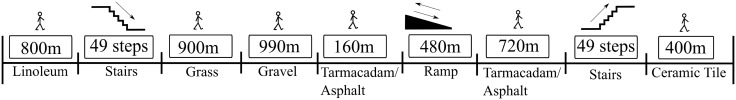
Prescribed walking route. Breakdown of surfaces and distances throughout the walking route.

Participants completed a prescribed flat walking route consisting of five different walking surfaces: linoleum (800m), natural lawn grass (900m), gravel (990m), ceramic tile (400m) and tarmacadam/asphalt (880m). In addition, the prescribed walking route included stair walking (49 steps up and 49 steps down, step height 16cm) and ramp walking (240m up and 240m down at an incline of 4.05%) (Fig 2).

Each participant completed the walking route twice: the first time wearing standard running shoes, the second time wearing hard-soled dress shoes. At all times the participants were asked to walk at their normal walking pace, in other words, self-selected walking speed.

### Inter-device Reliability

There were between three and five of each device type used during testing. Prior to testing, each unit of each device type was tested over a 400m flat walk of uniform ceramic tile surface while wearing hard-soled shoes. One investigator wore each unit of a device type simultaneously for the 400m walk. The step counts on each unit of that device type were then compared. There was found to be no difference in step counts between the Fitbit ^™^ units. The largest difference between the units of the remaining activity monitors was 0.19% for the NL-2000 ^™^ units, 1.55% for the ActivPAL ^™^ units, and 13.67% for the Garmin ^™^ units. For the Garmin units, any step counts outside of ±2 SD of the observed count were excluded from analysis.

### Statistical Analysis

All statistical analyses were carried out using SPSS (SPSS for Mac, version 20, IBM Corporation). Sample size was chosen by selecting a type I error rate (α) of 5%, a power (1—β) of 0.80, a sampling ratio of 1 (κ = n_A_ / n_B_), and a standard deviation of 250 steps (2.5% of the recommended daily step count of 10,000 steps). The Shapiro-Wilk test was used to analyze normality of data. Data were then fitted to a repeated measures model as this study followed a repeated measures design. Repeated-measures analysis of variance and post-hoc follow-up were used to detect differences in step count between the true step count extracted manually from the video recordings and the step count registered by each activity monitor and to detect step count detection errors associated with each surface type during the walking route. The Greenhouse-Geisser correction was used to correct any violations of the assumption of sphericity. The mean absolute percentage error (MAPE) was calculated for each activity monitor using Eq 1 with N the number of steps as extracted from video analysis and Ñ the number of steps as recorded from each activity monitor.

$$MAPE=\left[\frac{\text{Ñ}-N}{N}\right]*100$$ (1)

There is no standard for acceptable error in step detection in activity monitors, but we have chosen to select a 5% error zone as being acceptable. Bland-Altman plots were plotted to examine the agreement in step count between the observed step count and the step count recorded on the individual activity monitors. An unpaired t test was carried out to assess any differences in step count between participants classed as ‘fast’ and ‘medium to slow’ speed walkers.

## Results

**Fig 3:**
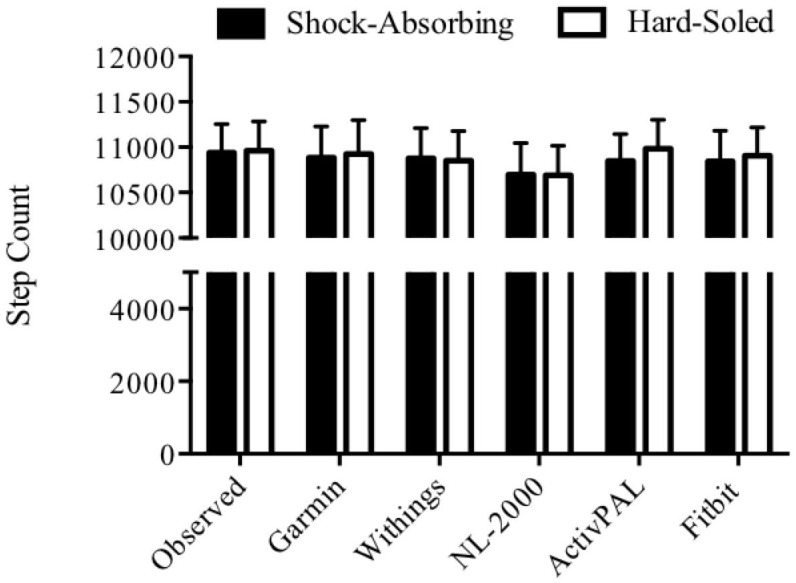
Overall walking route step count. Independent of surface type, there was no statistically significant difference in step count versus the observed step count or versus the ActivPAL ^™^ activity monitor. All data represent mean ± SEM.

Step counts obtained from video analysis are referred to as the observed step count and were compared to output counts from each activity monitor. Surface type did not affect step count detection on the four activity monitors tested. The mean step counts for the four activity monitors tested over each section of the walking route are given in Table 2. There was no statistically significant difference in activity monitor detected step count vs. the observed step count for the Withings Smart Activity Monitor Tracker (Pulse O_2_) ^™^, NL-2000 pedometer ^™^, Garmin Vivofit ^™^, or Fitbit One ^™^ activity monitors on the linoleum (P = 0.106), gravel (P = 0.131), natural lawn grass (P = 0.195), tarmacadam/asphalt (P = 0.286), or ceramic tile (P = 0.457) surfaces. There was also no statistically significant difference in activity monitor detected step count versus the observed count for the Withings Smart Activity Monitor Tracker (Pulse O_2_) ^™^, NL-2000 pedometer ^™^, Garmin Vivofit ^™^, and Fitbit One ^™^ activity monitors on the ramp section of the walking route (P = 0.591). Stairs walking did not affect step count, with no statistically significant difference in activity monitor detected step count vs. the observed step count for any activity monitor (P>0.05 for all). Over the entire walking route, independent of surface type, there was no statistically significant difference in step count between the observed count and the four activity monitors tested (P > 0.05; Fig 3). There was also no statistically significant difference in step count between the step count on the ActivPAL ^™^ reference device and the Withings Smart Activity Monitor Tracker (Pulse O_2_) ^™^, NL-2000 pedometer ^™^, Garmin Vivofit ^™^, or Fitbit One ^™^ activity monitors (P > 0.05 for all). There was no statistically significant difference in step count when the devices were compared to each other on any surface type or overall, independent of surface type (P > 0.05 for all). Mean absolute percentage errors for each of the four activity monitors are given in Table 3. Over the prescribed walking route, eight mean absolute percentage errors were found to be outside of our selected 5% error zone. The Withings Smart Activity Monitor Tracker (Pulse O_2_) ^™^ had a step count error for stair walking only, with errors on descending stairs of 6.64% and on ascending stairs of 5.93%. The Garmin ^™^ had step count errors on the ceramic tile of 5.43%, on ascending stairs of 11.33% and on descending stairs of 6.48%. The NL-2000 pedometer ^™^ step count error on ceramic tile was 7.66% and on ascending stairs of 8.65%. The Fitbit ^™^ had step count error on descending stairs of 6.03%.

**Table 2:** Mean step counts observed and registered on the four activity monitors tested over the different surface types of the walking route.

	Observed Step Count	Withings ^™^	Garmin ^™^	NL-2000 ^™^	Fitbit ^™^	P Value
Natural Lawn Grass	1937 ± 166	1945 ± 190	1921 ± 156	1888 ± 222	1928 ± 169	0.195
Gravel	2422 ± 158	2408 ± 143	2359 ± 150	2388 ± 251	2424 ± 159	0.131
Linoleum	1979 ± 182	1982 ± 193	1987 ± 175	1922 ± 268	1984 ± 191	0.106
Tarmacadam/ Asphalt	2234 ± 234	2186 ± 251	2185 ± 235	2214 ± 239	2210 ± 303	0.286
Ceramic Tile	469 ± 31	455 ± 50	465 ± 21	436 ± 108	473 ± 41	0.457
Ramp	954 ± 63	965 ± 97	951 ± 78	952 ± 87	959 ± 83	0.591
Stairs Up	111 ± 9	112 ± 8	114 ± 10	102 ± 19	110 ± 11	0.083
Stairs Down	117 ± 4	111 ± 14	115 ± 11	113 ± 9	118 ± 23	0.129
Total Step Count	10950 ± 1209	10866 ± 1246	10950 ± 1364	10695 ± 1281	10875 ± 1236	0.213

There was no statistically significant difference in step count between the observed step count and the step count registered on each activity monitor over the different surfaces tested. Figures represent mean ± SD.

**Table 3:** Mean absolute percentage error (MAPE) and error range for the four activity monitors tested irrespective of footwear type.

	Withings ^™^		Garmin ^™^		NL-2000 ^™^		Fitbit ^™^	
	MAPE	Error Range	MAPE	Error Range	MAPE	Error Range	MAPE	Error Range
Natural Lawn Grass	1.70	-6.34 to 15.96	3.99	-16.41 to 14.09	3.68	-25.95 to 7.41	1.24	-11.99 to 2.99
Gravel	1.81	-17.89 to 9.01	4.55	-16.29 to 11.67	2.83	-42.59 to 8.96	0.69	-1.68 to 4.70
Linoleum	1.32	-6.51 to 5.61	3.15	-9.54 to 7.65	4.17	-49.13 to 5.16	1.34	-7.78 to 7.20
Tarmacadam/ Asphalt	2.40	-23.58 to 3.05	4.66	-33.67 to 3.57	1.28	-6.84 to 3.13	3.26	-37.59 to 19.04
Ceramic Tile	3.20	-22.20 to 0.22	**5.43**	-16.89 to 13.23	**7.66**	-63.43 to 0.00	1.06	-1.04 to 7.02
Ramp	3.52	-29.69 to 22.53	4.10	-13.98 to 16.13	3.08	-13.76 to 22.00	2.18	-15.76 to 24.55
Stairs Up	**6.64**	-22.94 to 79.41	**11.33**	-14.29 to 80.88	**8.65**	- 47.71 to 2.86	2.93	-20.69 to 8.62
Stairs Down	**5.93**	-47.46 to 2.61	**6.48**	-10.53 to 5.58	4.43	-25.62 to 5.45	**6.03**	-16.36 to 99.16
Total Step Count	1.36	-9.55 to 3.42	4.61	-21.73 to 9.46	2.82	-18.61 to 3.13	1.44	-8.01 to 3.89

Eight mean absolute percentage errors were found to be outside of our selected 5% error zone (in bold): the Withings Smart Activity Monitor Tracker (Pulse O_2_) ^™^ step count error on the stairs down and stairs up sections, the Garmin ^™^ step count error on the ceramic tile and stairs up and stairs down sections, NL-2000 pedometer ^™^ step count error on the ceramic tile and stairs up sections, and the Fitbit ^™^ step count error on the stairs down section of the walking route. The error range shows the variation in percentage error for each activity monitor over each surface type.

All other mean absolute percentage errors remained within the 5% error zone. The absolute percentage error range is also is in Table 3. Although there was no significant difference in the step counts observed versus the step counts detected by the activity monitors, these values show the variation in the percentage error for the activity monitors (Table 3). For example, when walking down stairs, the Fitbit One ^™^ activity monitor had a mean absolute percentage error of 6.03% and a mean absolute percentage error range of -16.36% to 99.16%.

When all values lying ±2SD from the mean observed step count were removed, only two mean absolute percentage errors were found to be outside the 5% error zone (Table 4). These were both for the Garmin ^™^ device, with error on ascending stairs of 6.59% and on descending stairs of 5.03%.

**Table 4:** Mean absolute percentage error (MAPE) and error range for the four activity monitors tested: all values ± 2SD from the mean observed step count excluded.

	Withings ^™^		Garmin ^™^		NL-2000 ^™^		Fitbit ^™^	
	MAPE	Error Range	MAPE	Error Range	MAPE	Error Range	MAPE	Error Range
Natural Lawn Grass	1.21	-6.34 to 7.47	2.18	-14.31 to 5.19	2.23	-14.40 to 7.41	1.24	-11.99 to 2.99
Gravel	1.25	-5.88 to 9.01	2.75	-10.06 to 11.67	1.46	-7.17 to 8.96	0.69	-1.68 to 4.70
Linoleum	1.32	-6.51 to 5.61	3.15	-9.54 to 7.65	2.02	-8.07 to 5.16	1.34	-7.78 to 7.20
Tarmacadam/ Asphalt	1.67	-6.91 to 3.05	3.10	-14.45 to 3.57	1.28	-6.84 to 3.13	2.07	-8.33 to 19.04
Ceramic Tile	0.82	-1.46 to 0.22	1.36	-0.64 to 6.41	0.69	-1.60 to 0	1.06	-1.04 to 7.02
Ramp	1.91	-4.04 to 10.99	2.96	-13.98 to 7.28	2.43	-13.76 to 6.99	0.90	-4.36 to 4.11
Stairs Up	3.46	-13.04 to 14.02	**6.59**	-14.29 to 16.22	2.65	-8.04 to 2.86	2.32	-9.09 to 8.62
Stairs Down	2.20	-7.29 to 2.61	**5.03**	-7.27 to 5.88	1.91	-6.25 to 5.45	2.10	-7.14 to 1.65
Total Step Count	1.36	-9.55 to 3.42	4.58	-21.73 to 9.46	2.82	-18.61 to 3.13	1.44	-8.01 to 3.89

With all values lying ±2SD from the mean observed step count removed, only two mean absolute percentage errors were found to be outside of our selected 5% error zone (in bold): both for the Garmin ^™^ device, on the stairs up and stairs down sections. The error range shows the variation in percentage error for each activity monitor over each surface type.

**Fig 4:**
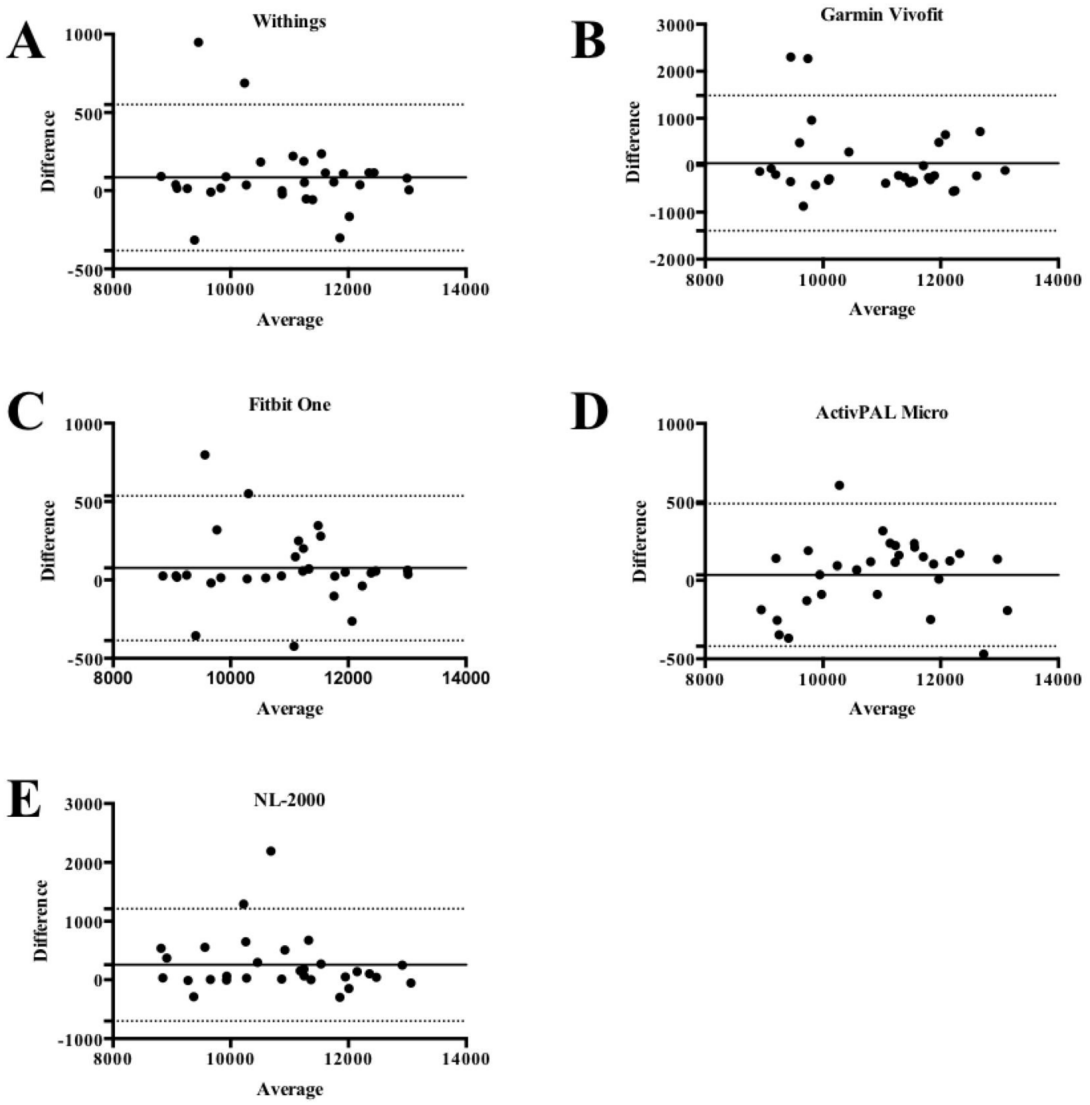
Bland Altman plots for step counts of the A. Withings ^™^, B. Garmin ^™^, C. Fitbit ^™^, D. ActivPAL ^™^, and E. NL-2000 ^™^ activity monitors. The solid line represents the mean step count difference between the step count observed from the video recordings and each activity monitor for the overall step count. The dashed lines indicate mean ± limits of agreement (1.96*SD).

Bland-Altman plots for the number of steps showed average ± limits of agreement underestimation of 84±238, 44±735, 75±235, 35±232, and 254±488 for the Withings ^™^, Garmin ^™^, Fitbit ^™^, ActivPAL ^™^, and NL-2000 ^™^ activity monitors respectively (Fig 4).

Footwear type did not affect step detection of any activity monitor; there was no statistically significant difference in step count between the observed count and any activity monitor (P>0.05 for all).

Participants took a median time of 4 hours and 4 minutes to complete the entire walking route. Seven participants were classed as being walkers of a ‘fast’ speed as they completed the walking route in a time of between 2 hours and 6 minutes and 3 hours and 2 minutes, a gap of 1 hour and 2 minutes from the median walking time. Eight participants were classed as being walkers of a ‘medium to slow’ speed as they completed the walking route in a time between the median time (4 hours and 4 minutes) and 4 hours and 38 minutes. There was a statistically significant difference in step count between the ‘medium to slow’ speed walkers and ‘fast’ speed walkers, with the ‘medium to slow’ speed walkers taking a greater number of steps (P = 0.002). Additionally, the mean walking speed for the ‘fast’ walkers was 0.84m/s compared to 0.74m/s for the ‘medium to slow’ speed walkers, a statistically significant difference (P = 0.031).

## Discussion

This study assessed four commercial activity monitors: the Withings Smart Activity Monitor Tracker (Pulse O_2_) ^™^, NL-2000 pedometer ^™^, Garmin Vivofit ^™^, and Fitbit One ^™^ for their step count detection accuracy over a prescribed walking route that reflected a range of surfaces that would typically be encountered to achieve the recommended daily step count and in a timeframe required to do so. In addition, we investigated two different types of footwear typically used throughout the day when aiming to achieve the recommended daily step count of 10,000 steps.

As stated by the WHO, physical activity includes a variety of activities, such as recreational or leisure-time physical activity, occupational (i.e. work), household chores, and transportation (e.g. walking or cycling) [5]. As such, the recommended daily step count of 10,000 steps would typically be achieved over a variety of different surfaces. Taking this into consideration, we tested a prescribed walking route consisting of various surfaces that would be encountered on a daily basis, namely linoleum, natural lawn grass, gravel, tarmacadam/asphalt, and ceramic tile surfaces. All activity monitors were found to be accurate in step detection, irrespective of surface type. This is an important finding as step count is one of the main parameters utilized in physical activity quantification. It is vital that persons changing their behavior to adhere to public health physical activity recommendations can be confident in the sensitivity of the activity monitors they are employing to achieve their physical activity goals and reach the recommended daily step count of 10,000 steps. Additionally, our results correspond with those obtained by Brown et al. when they investigated the accuracy of the ActiPed ^™^ activity monitor over two different surface types, namely grass and concrete. Participants walked 1,010m on grass and 1,070m on concrete, with the authors finding the ActiPed ^™^ to be accurate in step count detection regardless of surface type [36].

When considering step counts, as opposed to surface type, participants in our study exceeded the recommended daily step count of 10,000 steps, taking an average of 10,950 steps to complete the prescribed walking route. Over the course of the entire walking route, independent of surface type, all activity monitors were found to be sensitive in their step detection accuracy, on both flat surfaces and stairs walking. Indeed, it is important to consider the effect of stairs walking, which is an activity of daily living and part of the normal daily step count goal for many adults. We observed similar results to those obtained by Storm et al., who investigated the sensitivity of both the ActivPAL ^™^ and Movemonitor ^™^ activity monitors during stairs walking at a self-selected walking speed, finding both activity monitors to be accurate in step detection [19]. Accuracy is an important factor to consider when the quantification of physical activity is paramount, such as when physical activity may be prescribed as an intervention in the treatment or prevention of a disease state. Sensitivity in this case is the probability that a step will be detected when a step is actually performed and a measure of all true positives (TP; i.e. a step was performed and it was detected) divided by the true positive (TP) plus the false negatives (FN; i.e. a step was performed but not detected). Due to the nature of the data output from the activity monitors, calculating FN is not possible thus we presented the MAPE as a valid measure of accuracy.

When we investigated the effect of two different types of footwear: running shoes and hard-soled dress shoes, on the sensitivity of the activity monitors, we found no effect on step detection sensitivity. To the best of our knowledge, this is the first study to assess the effects of footwear on the step detection sensitivity of activity monitors and is an important and positive finding, as activities carried out to achieve the recommended step count of 10,000 will most likely be done in various different types of footwear. However, we only investigated two different types of footwear. It would also be of interest to investigate other types of footwear that would often be worn on a daily basis when accumulating the recommended daily step count of 10,000 steps. Examples include slippers, flip-flops, high-heeled shoes, and hiking boots. These different types of footwear differ in terms of their sole type, degree of foot contact with the ground/floor, and the way in which they are worn, i.e. loose fitting or tight fitting on the foot, all of which may play a role in the step detection sensitivity of activity monitors.

The authors do recognize that limitations exist with our study, namely we have tested the accuracy of four commercial activity monitors but have not evaluated their specificity. For example, it is also of vital importance to validate activity monitors during a variety of activities of daily living that do not involve direct stepping, such as driving, cycling, and swimming. Furthermore, acceleration signals recorded by activity monitors can differ depending on the location of attachment [37]. Thus, the attachment location for each activity monitor is important to consider. In this study, we assessed each activity monitor in only one location. However, we specifically chose attachment locations for each activity monitor that was specified by the manufacturers.

Additionally, it is important to consider walking speed when evaluating the sensitivity of activity monitors. Slow walking speeds, for example, have been shown to reduce the sensitivity of some activity monitors [18, 27, 38–40]. In this study, we evaluated different distributions of walking speeds within the normal walking self-selected walking speeds and found a greater speed and lesser number of steps in the ‘fast’ speed walkers. However, evaluation on a range of specific walking speeds, such as on a treadmill, would be of benefit to further elucidate the effect of walking speed on activity monitor step detection sensitivity.

### Conclusions

Our study provides a comprehensive evaluation of four commercial activity monitors when (i) walking on different surfaces that reflect the range of surfaces that will typically be used to achieve the daily step count goal, (ii) walking with different footwear that reflects the different types of footwear expected to be used throughout the day, and (iii) walking for periods of time that properly reflect the time required to achieve the recommended daily step count. All activity monitors tested were accurate in their step detection sensitivity and are valid monitors for physical activity quantification over the variety of different flat surfaces tested and when stairs and ramp walking, when wearing both running shoes and hard-soled dress shoes, and over a timeframe necessary for accumulating the recommended daily step count of 10,000 steps. It is important to consider accuracy for activity monitors, particularly when physical activity in the form of stepping activities is prescribed as an intervention in the treatment or prevention of a disease state. All aspects of daily physical activity that go towards achieving the daily recommended step count of 10,000 steps are necessary to consider in accurate physical activity quantification.
